# Can computational fluid dynamics simulations predict a distal stent graft-induced new entry after frozen elephant trunk operation?

**DOI:** 10.3389/fcvm.2025.1671628

**Published:** 2025-10-03

**Authors:** Anja Osswald, Konstantinos Tsagakis, Matthias Thielmann, Sharaf-Eldin Shehada, Rolf Alexander Jánosi, Payam Akhyari, Christof Karmonik

**Affiliations:** ^1^ Department of Thoracic and Cardiovascular Surgery, West-German Heart and Vascular Center Essen, University Duisburg-Essen, Essen, Germany; ^2^ Department of Cardiology, West-German Heart and Vascular Center Essen, University Duisburg-Essen, Essen, Germany; ^3^ Translational Imaging Center, MRI Core, Houston Methodist Research Institute, Houston, TX, United States

**Keywords:** wall shear stress, computational fluid dynamics simulation, distal stent graft-induced new entry, frozen elephant trunk, aortic dissection

## Abstract

**Objectives:**

Distal stent graft-induced new entry (dSINE) is a complication after frozen elephant trunk (FET) procedure. The purpose of this study was to identify the hemodynamic profile of the aorta associated with dSINE development using computational fluid dynamics (CFD) simulation.

**Methods:**

30 patients, 15 who had developed a dSINE (dSINE group) and 15 without any further vascular events (control group), undergoing an FET operation for aortic dissection were retrospectively included in this CFD study. Patient-specific 3D surface models of the aortic lumen were reconstructed from computed tomography angiographic (CTA) datasets, utilizing the CTA acquired before dSINE onset. Steady-state CFD simulations were performed with laminar blood flow and zero-pressure outlet conditions to assess velocity magnitudes, wall shear stress (WSS), and vorticity within the stent graft (SG), its distal landing zone and further downstream.

**Results:**

In the dSINE group, WSS was significantly elevated distal to the SG compared to WSS within the SG and at its landing (2.95 ± 1.47 Pa vs. 1.56 ± 0.71 Pa and 2.00 ± 0.81 Pa, *p* < 0.001 for both comparisons). In the control group, this distinct pattern of distal WSS elevated distal to the SG in comparison to other locations was not observed. Similarly, vorticity increased significantly distally to the SG in the dSINE group, but not in the control group.

**Conclusions:**

Increased WSS distal to the SG compared to within the SG and its landing zone seem to be associated with dSINE development. CFD can be a useful tool to understand SG-induced hemodynamic changes in the aorta to help predict complications after FET.

## 1 Introduction

The frozen elephant trunk (FET) procedure is a well-established surgical treatment for acute and chronic type-A aortic dissections (AD), combining open surgical and endovascular approaches. It has demonstrated favorable outcomes in promoting positive aortic remodeling, particularly in the downstream aorta (1). However, adverse events such as aneurysmal growth, rupture and degeneration of residual dissection and a distal stent graft-induced new entry (dSINE) represent the major cause of mid- and long-term clinical complications (1). dSINE refers to a new tear in the intimal layer of the aortic wall caused by the stent graft (SG), excluding factors like natural disease progression or iatrogenic procedural injury (2). This tear often causes an antegrade perfusion of the false lumen (FL), posing a significant risk for negative aortic remodeling and subsequent rupture (3). Therefore, dSINE necessitates an additional aortic reintervention, most commonly endovascular treatment. Incidences after FET (12.7%) are more frequent than thoracic endovascular repair, for which the rate is approximately 5% (4, 5). These rates also vary depending on the type of FET prosthesis used (6). Risk factors for dSINE include chronic AD, excessive oversizing ratio, less taper ratio, sharp angle between the SG and true lumen (TL) and the total aortic lumen at the SG landing zone (4, 7–9). However, many retrospective studies show no significant differences in the underlying disease, aortic features, or SG size between patients with or without dSINE (10, 11).

Despite extensive clinical and anatomical research, no reliable predictors of dSINE have been identified. While medical imaging provides detailed anatomical and physiological insights, computational fluid dynamics (CFD) analysis extends this understanding by visualizing complex flow patterns and hemodynamics. Patient-specific reconstruction of the aorta from CT or magnetic resonance imaging (MRI) data forms the basis for advanced simulations using CFD and fluid–structure interaction methods. These approaches allow for detailed, non-invasive assessment of blood flow, velocity fields, pressure distribution, and wall shear stress—the tangential force exerted on the endothelial lining of the arterial wall (12). These parameters are critical for comprehending vascular stresses and their role in aortic remodeling and potential complications. The foundation for such simulations lies in precise medical image processing, typically based on contrast-enhanced computed tomography angiography (CTA) or MRI. Segmentation techniques are used to extract vascular structures and generate three-dimensional surface models, which serve as input for numerical simulations. Advances in segmentation algorithms, surface reconstruction, and mesh generation have significantly improved the fidelity and reproducibility of CFD models in clinical research. Recent work has demonstrated that such simulations can reliably reproduce hemodynamic phenomena observed *in vitro*, including complex flow patterns and pressure differences related to tear morphology in aortic dissections (13). In parallel, clinical applications of CFD have shown that altered hematocrit levels and flow conditions substantially influence WSS and thrombus formation in the FL, thereby highlighting the potential of CFD to predict long-term complications after thoracic endovascular aortic repair (14). Together, these developments underline the value of combining advanced image processing with computational hemodynamics for improving risk stratification and personalized follow-up in patients with aortic dissection.

The aim of this study was to identify hemodynamic risk factors associated with the development of dSINE by analyzing the aortic hemodynamic profile before its occurrence. Comparing the hemodynamic patterns of patients who developed dSINE with those in a control group, we aimed to uncover specific differences in blood flow dynamics and vascular stresses that could potentially aid in predicting the risk of dSINE development in the future.

## 2 Methods

### 2.1 Study design

**Figure 1:**
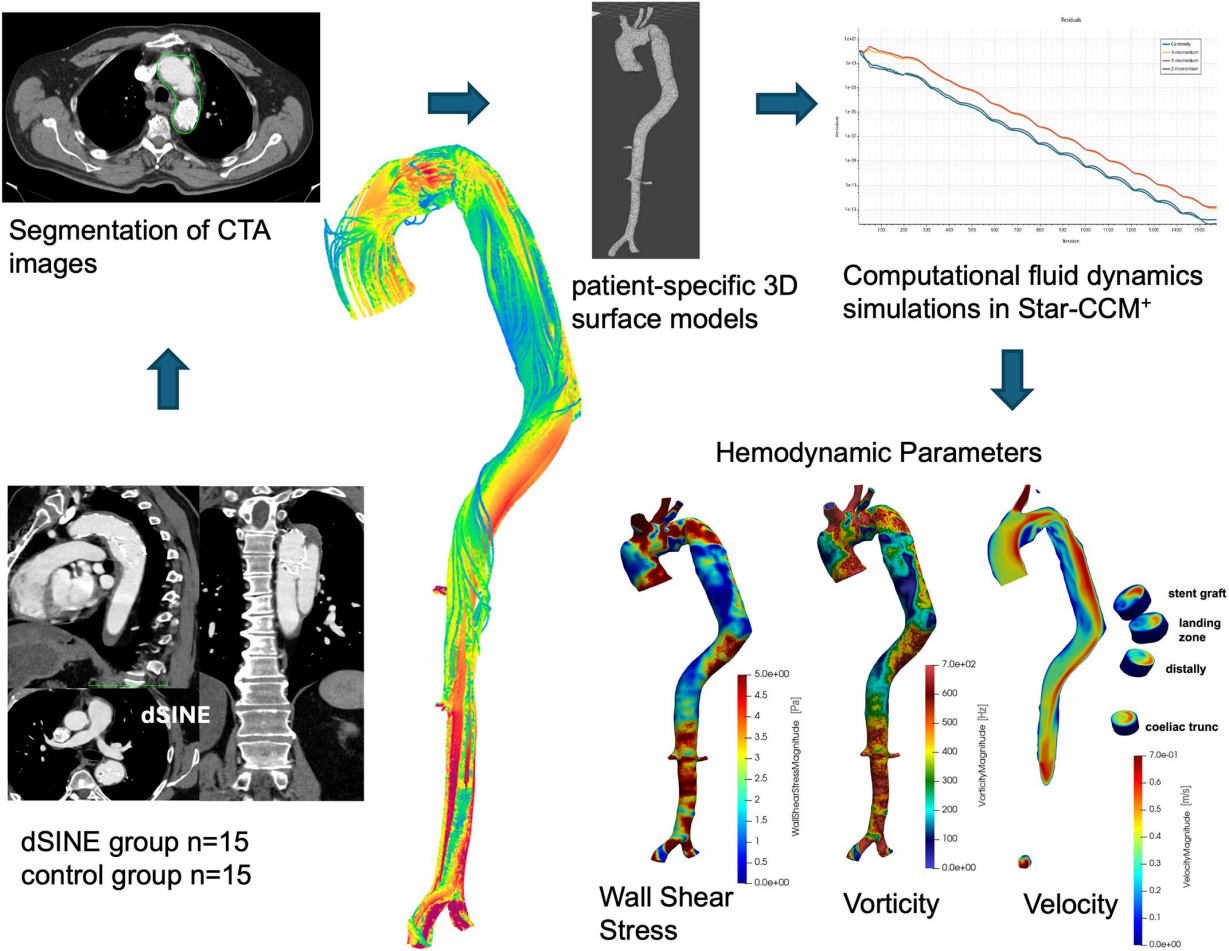
The CFD workflow begins with CTA segmentation and creation of patient-specific 3D aortic models. These models are used in Star-CCM+ to calculate hemodynamic parameters such as WSS, vorticity, and velocity at key aortic locations, including the stent graft, landing zone, distal aorta, and celiac trunk.

The study was approved by institutional ethics board of the University Duisburg-Essen (No. 22-10561-BO) on February 23, 2022, with a waiver of individual consent. Between 2006 and 2022, 332 FET procedures were performed for acute or chronic AD at our institution, with postoperative CTA imaging available in 296 cases. Among these, 18 patients (6.1%) developed a dSINE, defined as a new intimal tear caused by the SG of the FET prosthesis, during follow-up (2, 15). After applying inclusion criteria requiring a high-quality CTA prior to dSINE onset, 15 patients were eligible for analysis and included in this single-center CFD study as the dSINE group. For these patients, the last CTA scan before the development of dSINE was used for CFD simulations as hemodynamic risk factors were expected to be most prominent at this time point (mean time between surgery and CTA used for the simulations: 1.65 ± 1.48 years). For the control group 15 patients who had no further vascular events, such as dSINE, endoleaks or a second downstream procedure (planned or unexpected) for at least 5 years after the initial FET operation were randomly selected. The follow-up CTA images with the best quality were chosen for CFD simulations (mean time from surgery to CTA used for simulations: 3.27 ± 3.20 years). CFD simulations were then conducted using the CTA data from both groups to compare hemodynamic parameters between patients with and without dSINE (Figure 1).

### 2.2 Surgical procedure

FET surgery was performed with the E-vita open prothesis (Artivion, Hechingen, Germany) for either acute or chronic AD (16). This hybrid procedure combines open surgical aortic replacement with endovascular stent grafting, addressing both the proximal and distal aorta. FET SG sizing aimed for a 1:1 adaptation to the maximum TL diameter and was otherwise limited to a maximum of 10% of oversizing. Reinterventions for dSINE were performed endovascularly, with proximal TEVAR sizing aimed at 10% oversizing and 3–5 cm overlap with the FET SG.

### 2.3 Aortic measurements

**Figure 2:**
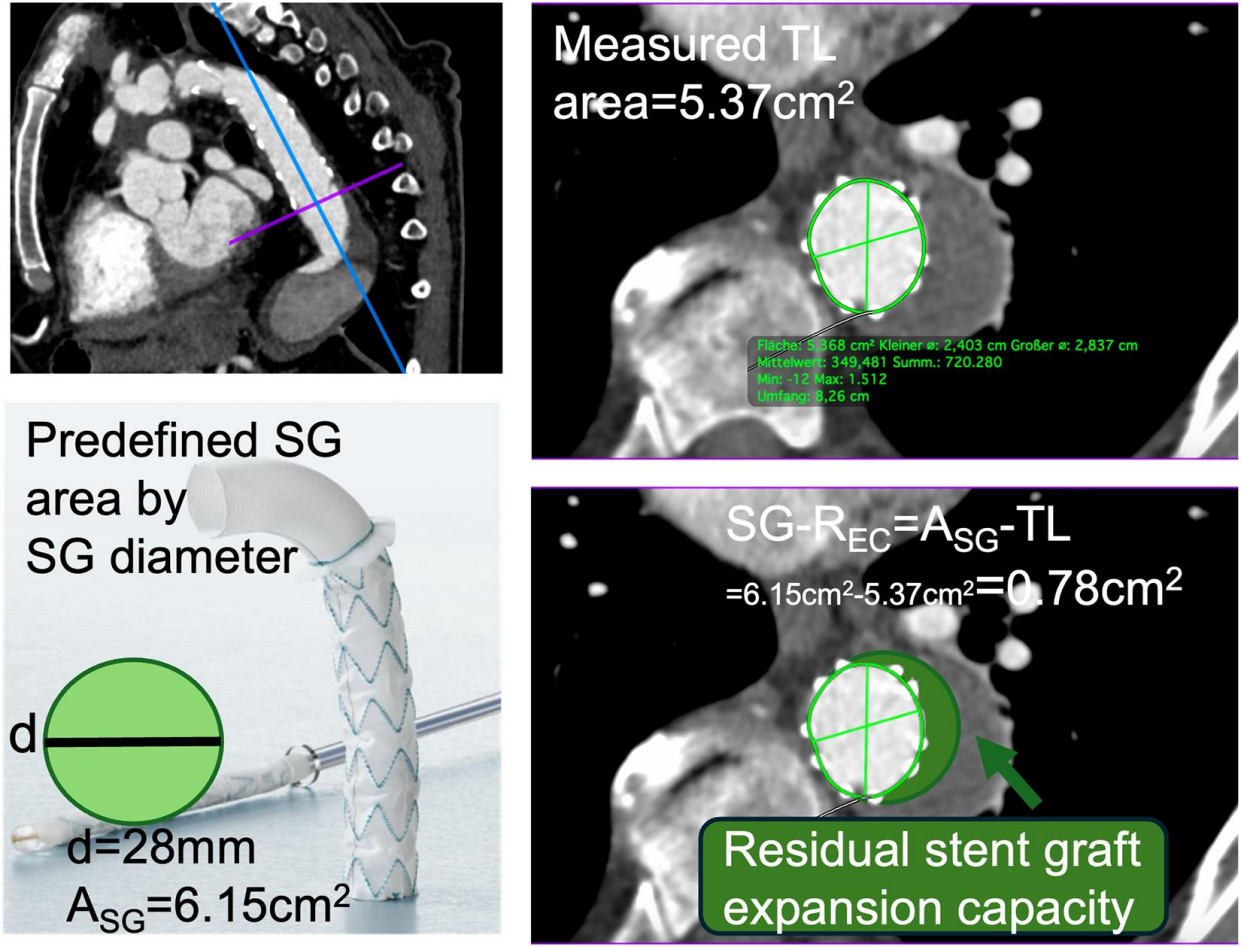
CTA with multiplanar reconstruction illustrates the measurement plane at the distal stent graft (SG) landing zone. The cross-sectional image shows the measured true lumen (TL) area, which is compared with the predefined (SG) area derived from the device diameter. SG-REC is calculated as the difference between these two areas (SG-REC = ASG—TL) and reflects the residual expansion potential of the stent graft after deployment.

Aortic areas (cm²) at the landing zone were measured using OsiriX (Pixemo Sarl, Bernex, Switzerland, version 14.0) using double oblique 3D multiplanar reconstruction, perpendicular to the centerline. To quantify the degree of SG unfolding after deployment, the difference between the predefined fabricated SG lumen area and the measured TL area was calculated. This difference was, as previously described, defined as the “SG residual expansion capacity” (SG-R_EC_), (Figure 2), (9).

### 2.4 CTA image pre-processing—patient-specific aorta model

Thin-sliced triple-phase CTA scans were used to reconstruct patient-specific 3D aortic geometries from the arterial phase using a grayscale threshold method with manual corrections for key anatomical features, including the dissection membrane.

The 2D aortic lumen surface was imported into a 3D modeling software (Meshmixer, Autodesk, version 3.5.474). The region of interest comprising the ascending aorta, the aortic arch including the supra-aortic vessels, the descending aorta with the SG and the iliac arteries was isolated and the corresponding surface was smoothed by adaptive remeshing. In a subset of cases (2 dSINE and 3 controls), the reconstruction extended only to just before or shortly beyond the coeliac trunk due to the limited availability of CT scans.

### 2.5 Computational fluid dynamics simulations

CFD simulations were performed using Star-CCM+ (Siemens CD-adapco, version 17.06.007-R8). Polyhedral meshes with a mean mesh size of 783,411 ± 575,382 elements were created from the imported 3D aortic geometries. Mesh-independence in these kinds of simulations was assessed in prior work by our group (17). Simulations of aortic dissection models with 170,000, 250,000, 320,000, and 420,000 elements were compared by analyzing mean, maximum, and standard deviation of velocity. Differences were <2.5% between the coarsest and finest meshes and <0.5% between medium and fine meshes, indicating that medium-resolution meshes were sufficient (17). In the present study, we employed meshes of approximately 780,000 elements on average, consistent with these validated criteria. Although the total mesh size varied due to CTA quality and scan extent, mesh settings were standardized across all models to ensure comparable accuracy of velocity, WSS, and vorticity estimation. Blood flow was modeled using the Navier-Stokes equations, which were solved using the finite volume method. Blood was modeled as a Newtonian fluid (dynamic viscosity 0.004 Pa*s, density 1,050 kg/m^3^) and a rigid wall model was employed. The Newtonian assumption is justified because, in large vessels such as the thoracic aorta, high shear rates render non-Newtonian effects on velocity fields and WSS negligible. The rigid wall model was chosen to reduce computational complexity and was considered suitable due to the increased aortic stiffness in patients with chronic dissection and SGs. This approach is consistent with recent CFD studies, which report that rigid wall assumptions are commonly applied and provide reliable estimates of key hemodynamic metrics such as WSS in AD settings (18). Due to the retrospective design of the study, direct flow or pressure measurements of inflow conditions were not available. Inflow was therefore set at a constant cardiac output with laminar flow and a velocity of 0.4 m/s approximating the average normal flow velocity in the ascending aorta. Consequently, all hemodynamic findings are largely attributed to the individual geometries of the aorta in each patient. A steady-state segregated flow solver with second-order accuracy was applied. Zero pressure conditions were applied at outlets, with extensions to the ascending aorta and major branches ensuring developed flow and minimizing boundary effects. The use of constant inflow and zero-pressure boundary conditions represents a simplification of physiologic hemodynamics but were chosen to reduce computational demands. Although this may affect the accuracy of absolute values, steady-state simulations can reproduce flow distributions in the thoracic aorta, and the present study focused on relative differences between patient groups rather than absolute quantification (19). Convergence was defined by residuals falling below ^10−5^ for continuity and momentum equations, and simulation runs were stopped once global flow parameters such as volume flow rate and pressure drop had stabilized.

### 2.6 Hemodynamic analysis

Velocity, WSS, and vorticity, were calculated as quantitative measures and visualized using Paraview (Kitware Inc.), as previously described (20). Measurements were performed within the SG, its landing zone, 2 cm distal to the SG end within the TL and FL and above the coeliac trunk (TL, FL). Hemodynamic parameters at the FET SG landing zone were measured separately at the inner and outer aortic wall curvatures to capture flow variations.

### 2.7 Statistical analysis

Statistical analyses were performed using SPSS (IBM SPSS Statistics for Windows, Version 29.0, Armonk, NY, USA: IBM Corp). Continuous variables were presented as mean ± standard deviation (SD), and categorical variables as percentages. Data normality was assessed with the Shapiro–Wilk test. For continuous variable comparisons, *t*-tests (paired or unpaired) were used for normal data, and Mann–Whitney U or Wilcoxon signed-rank tests for non-normal data.

## 3 Results

### 3.1 Baseline patient characteristics and aortic areas

dSINE occurred on average 2.84 ± 2.3 years after the initial FET procedure. In the dSINE group, most patients (80%) initially presented with chronic aortic dissection. Patient characteristics for both groups are summarized in Table 1, showing no significant differences in regard to the implanted FET prothesis (E-vita Open generation or SG length), the location of the anastomosis or the distal SG landing zone. The mean SG diameter was also similar, with 26.26 ± 2.25 mm in the dSINE group and 25.93 ± 3.03 mm in the control group.

**Table 1:** Patient characteristics.

Mean ± SD, *N* (%)	dSINE	Control	*p*-value
	*N* = 15 (%)	*N* = 15 (%)	
Demographics			
Age	58.7 ± 8.3	49.8 ± 10.1	0.008
Male	13 (86.7)	12 (80.0)	1.000
Connective tissue disorder	0 (0)	2 (13.3)	0.164
Disease			0.096
Acute AD	3 (20.0)	8 (53.3)	
Chronic AD	12 (80.0)	7 (46.7)	
Previous aortic surgery	4 (26.7)	9 (60.0)	0.096
Hypertension	14 (93.3)	13 (86.7)	1.000
FET characteristics			
Stentgraft generation			1.000
E-vita open	2 (13.3)	2 (13.3)	
E-vita open plus	13 (86.7)	13 (86.7)	
SG anastomosis			0.334
Zone 3	4 (26.7)	2 (13.3)	
Zone ≤2	11 (73.3)	13 (86.7)	
Stentgraft diameter, mm	26.26 ± 2.25	25.93 ± 3.03	0.628
Stentgraft length, mm			1.000
≤130	2 (13.3)	2 (13.3)	
≥150	13 (86.7)	13 (86.7)	
SG distal landing zone			0.334
Th5–7	7 (46.7)	12 (80.0)	
Th8–9	8 (53.3)	3 (20.0)	

### 3.2 Aortic areas

Table 2 presents the aortic measurements at the distal landing zone from both postoperative CTA images and scans used for the CFD simulations. The dSINE group had a significantly larger aortic area both postoperatively (11.67 ± 4.30 cm^2^ vs. 7.07 ± 2.88 cm^2^, *p* = 0.002) and before dSINE (13.09 ± 5.96 cm^2^ vs. 6.77 ± 3.17 cm^2^, *p* = 0.001) compared to the control group. Similarly, the FL area was significantly larger in the dSINE group at both time points, whereas the TL area was comparable between groups.

**Table 2:** Aortic measurements.

Mean ± SD	dSINE	Control	*p*-value
Distal landing zone postoperatively			
Aortic area, cm^2^	11.67 ± 4.30	7.07 ± 2.88	0.002
True lumen area, cm^2^	3.63 ± 0.92	3.91 ± 1.18	0.479
False lumen area, cm^2^	8.12 ± 4.78	3.14 ± 3.02	0.002
SG-R_EC_, cm^2^	1.77 ± 0.94	1.43 ± 0.86	0.313
Distal landing zone pre dSINE/control used for CFD simulations			
Aortic area, cm^2^	13.09 ± 5.96	6.77 ± 3.17	0.001
True lumen area, cm^2^	4.77 ± 1.00	4.54 ± 1.37	0.593
False lumen area, cm^2^	8.32 ± 6.08	2.24 ± 3.49	0.002
SG-R_EC_, cm^2^	0.68 ± 0.69	0.81 ± 0.89	0.659

SG-R_EC_: residual stent-graft expansion capacity.

The postoperative SG-R_EC_ was slightly larger in the dSINE group (1.77 ± 0.94 cm^2^) compared to the control group (1.43 ± 0.86 cm^2^, *p* = 0.313). By the time of the pre-dSINE measurements, the SG-R_EC_ in the dSINE group had decreased (0.68 ± 0.69 cm^2^) reaching a level similar, even slightly lower than, the control group (0.81 ± 0.89 cm^2^, *p* = 0.659). This suggests that greater SG unfolding may have occurred in the dSINE group over time.

### 3.3 Hemodynamic findings from CFD-simulations

#### 3.3.1 Wall shear stress

**Figure 3:**
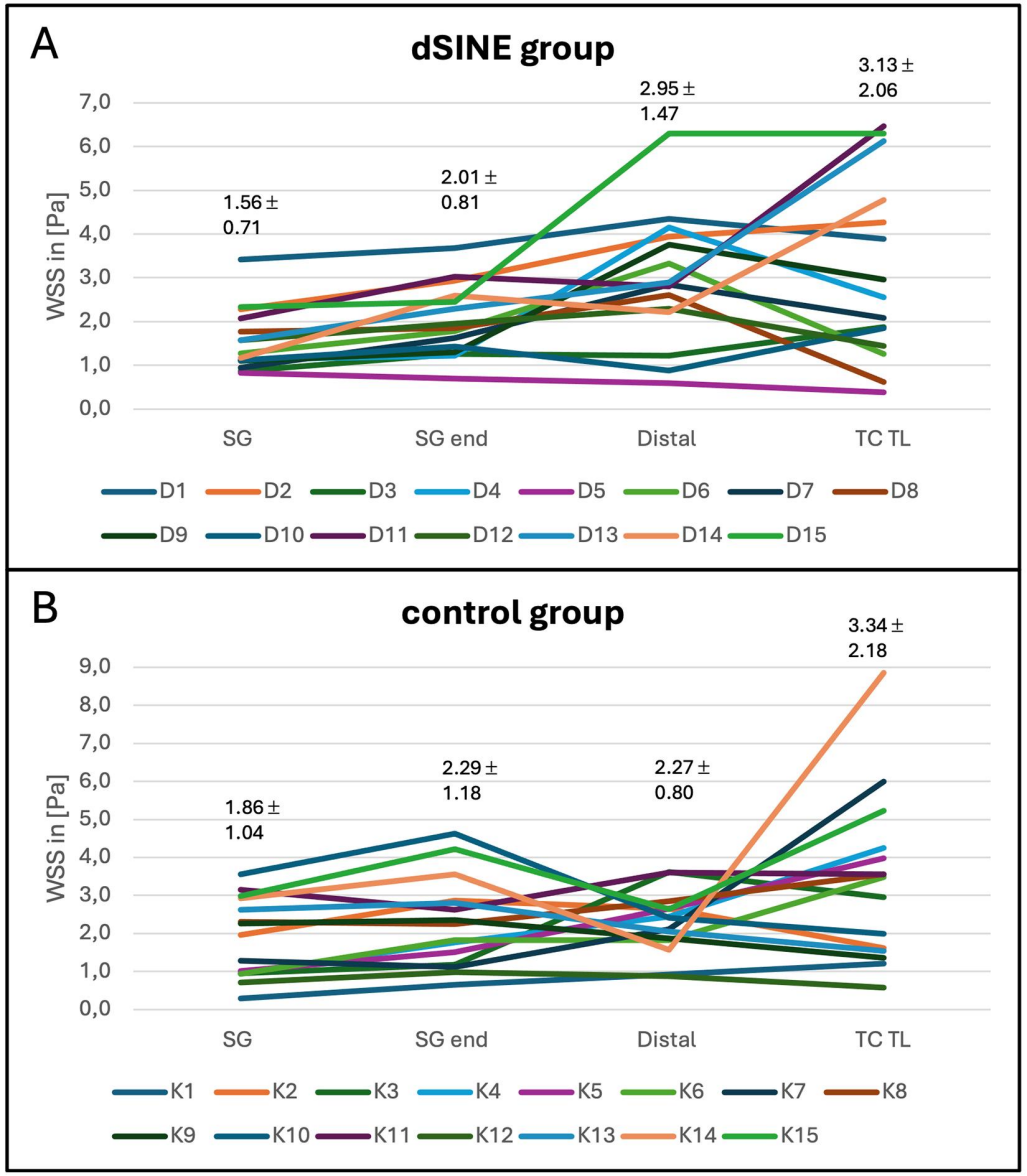
WSS measurements for individual patients, as well as mean values in the dSINE group **(A)**, (D1–D15) and control group **(B)**, (K1–K15) across the different aortic regions: SG, SG end, distally and in the true lumen at the level of the coeliac trunk (TC TL). These profiles illustrate the distinct WSS patterns between groups, with the dSINE group exhibiting an increase of WSS distally compared to within the SG and its landing zone.

CFD simulations revealed a distinct WSS distribution across the different segments in the aorta prior to dSINE development. In the dSINE group WSS was significantly elevated distal to the SG compared to WSS in the SG itself and at its landing zone (2.95 ± 1.47 Pa vs. 1.56 ± 0.71 Pa and 2.00 ± 0.81 Pa, *p* < 0.001 for both comparisons). Figure 3A displays the WSS for each patient in the dSINE group, as well as the mean values within the TL. While the WSS values vary between the patients, the increase distally to the SG is consistent across the majority of patients, suggesting a pattern where the distal aorta experiences increased shear stress compared to the neighboring areas.

This distinct WSS pattern was not observed in the control group, where no significant elevation in WSS was detected distal to the SG (2.27 ± 0.80 Pa) compared to within the SG (1.86 ± 1.04 Pa) or its landing zone (2.28 ± 1.18 Pa), (*p*-values: 0.140 and 0.910, respectively), (Figure 3B). The control group exhibits a more stable WSS profile across the entire aorta.

In both groups WSS was higher at the outer curvature compared to the inner curvature (dSINE group: 2.36 ± 0.94 vs. 1.66 ± 0.75; *p* = 0.005; control group 2.26 ± 1.33 vs. 1.77 ± 0.66; *p* = 0.041, respectively). The FL exhibits the lowest WSS, due to lower blood velocities. Distal to the SG WSS in the FL was 0.07 ± 0.06 Pa vs. 2.58 ± 1.67 Pa (*n* = 3, *p* = 0.109) in the TL in the dSINE group and 0.14 ± 0.28 Pa vs. 1.88 ± 0.72 Pa (*n* = 4, *P* = 0.068), respectively in the control group. At the level of the coeliac trunk the difference reached statistical significance in both groups (dSINE group: FL: 0.62 ± 1.03 Pa vs. TL: 3.64 ± 2.21 Pa; *p* = 0.007; control group: FL 0.74 ± 1.21 Pa vs. TL: 4.42 ± 2.86 Pa; *p* = 0.005).

**Figure 4:**
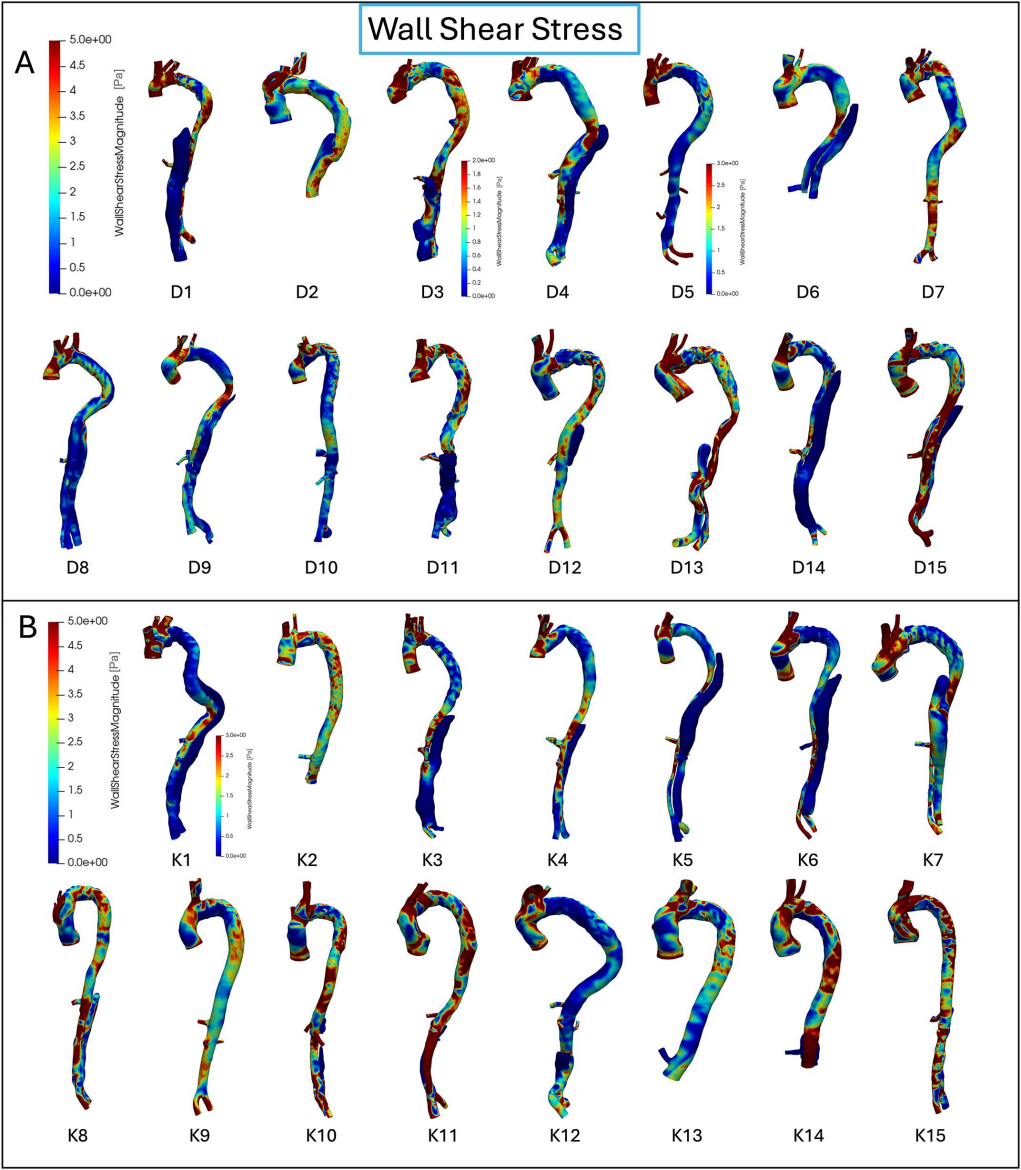
WSS distribution for each individual patient in the dSINE **(A)** and control group **(B)** the dSINE group exhibits elevated WSS, particularly in distal to the stent graft regions, while the control group shows more uniform and stable WSS profiles. Colour bars represent WSS magnitude (Pa), with red indicating high and blue low values.

The mean WSS distally to the SG was observed to be numerically higher in the dSINE group compared to the control group, albeit not reaching statistical significance (2.95 ± 1.47 Pa vs. 2.27 ± 0.80 Pa, *p*-value = 0.106). At all other locations, the mean WSS displayed slightly higher values in the control group; however statistically insignificant. This implies that while both groups may experience similar average shear stress levels, the distribution of WSS varies, with the dSINE group showing a more pronounced concentration of higher WSS distally to the SG (Figure 4).

#### 3.3.2 Velocity

**Figure 5:**
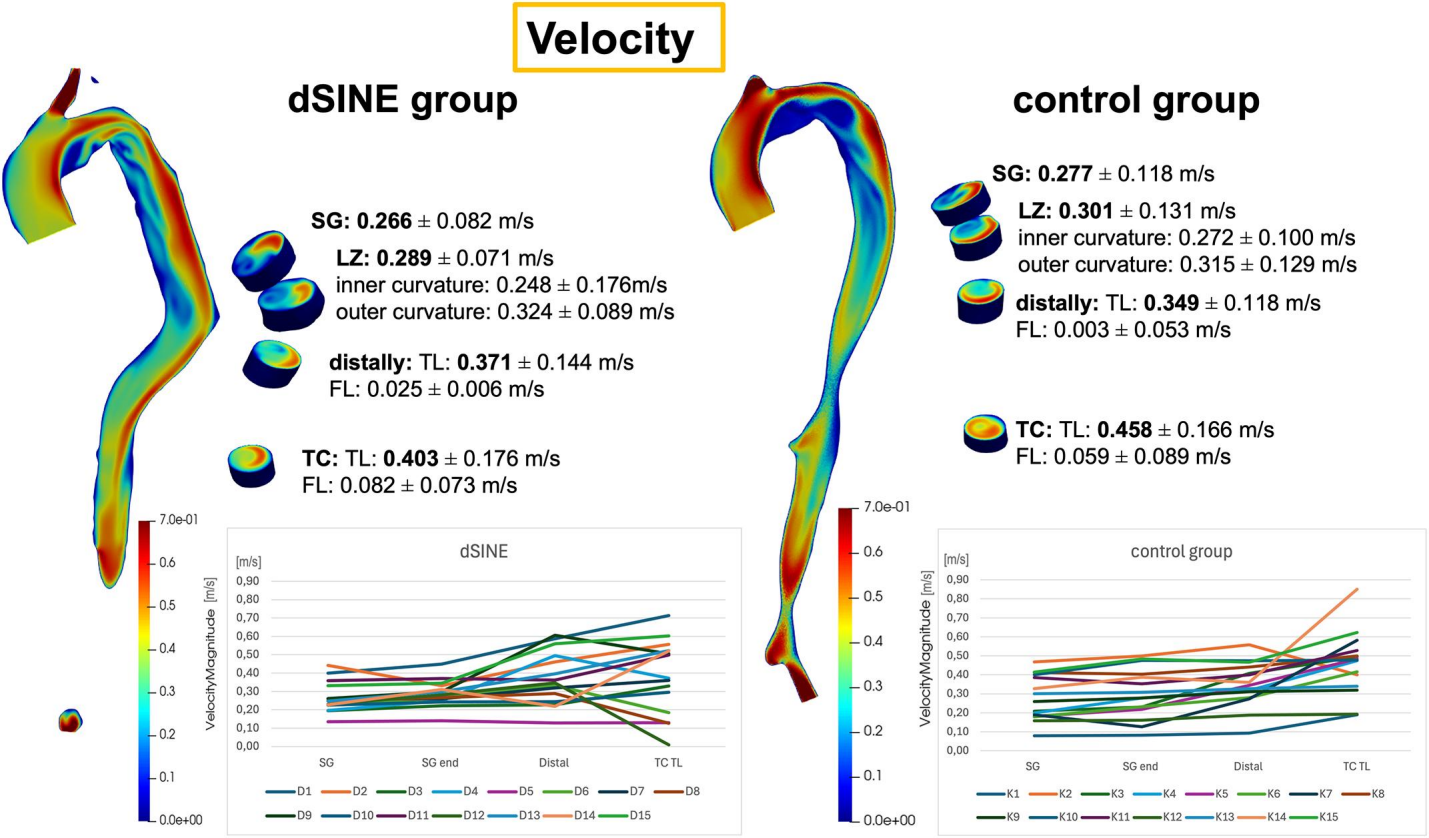
Velocity magnitude distributions are displayed for the dSINE (left) and the control group (right), with mean velocities and standard deviations across the aortic regions. The graphs show the values for each patient, demonstrating a more stable and consistent velocity pattern in the control group, while the dSINE group exhibits greater variability, particularly in the distal native aorta.

Velocities in the SG region in both groups are relatively low and consistent (0.266 ± 0.082 m/s in the dSINE group vs. 0.278 ± 0.118 m/s in the control group), indicating stabilized flow within the SG. In the most dSINE patients, velocities increased in the native aorta distally to the SG, ranging from 0.129–0.605 m/s (Figure 5). Mean velocity distal to the SG in the dSINE group was slightly higher compared to the control group (0.371 ± 0.144 m/s vs. 0.349 ± 0.118 m/s, respectively, *p* = 0.652). Overall, the control group demonstrated a more uniform velocity pattern across the aorta, reflecting stable, physiological blood flow, while the dSINE group displayed greater variability in flow velocities in the native aorta.

#### 3.3.3 Vorticity

**Figure 6:**
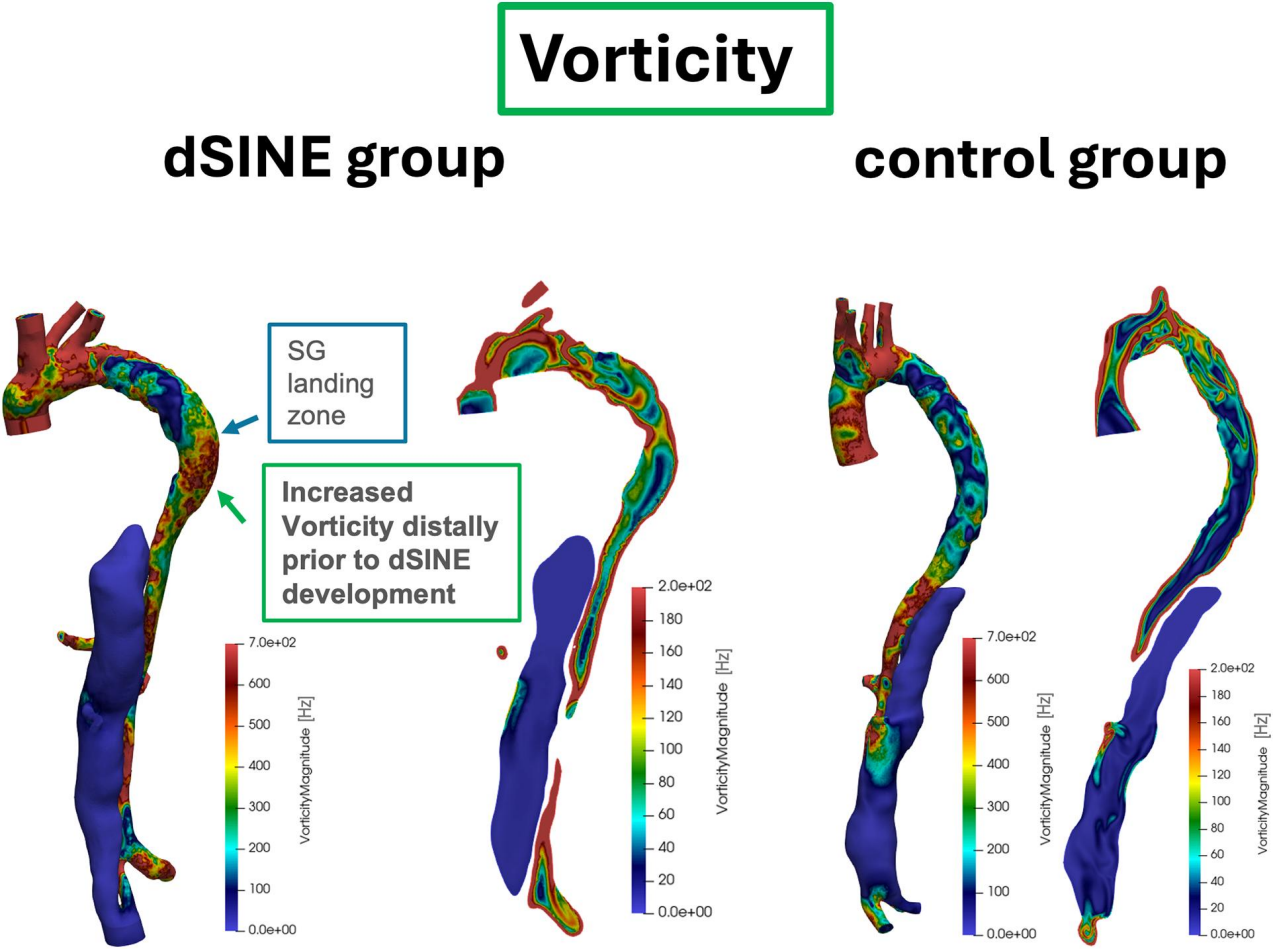
Vorticity patterns in the aorta for two patients from the dSINE group (left) and two from the control group (right). Vorticity display the same pattern observed in WSS, with an increase distal to the SG in the dSINE group, indicating a more disturbed flow environment, while the control group demonstrated a stable flow profile.

Vorticity showed the same pattern of a significant elevation distally to the SG compared to within the SG and at its landing zone in the dSINE group (144.75 ± 77.15 Hz vs. 96.98 ± 43.60 Hz, *p* = 0.031, and 98.60 ± 29.83 Hz, *p* = 0.013), respectively, (Figure 6). In the control group there was no significant increase in vorticity distally to the SG comared to within the SG or to its landing zone (111.75 ± 36.12 Hz vs. 99.07 ± 42.26 Hz, *p* = 0.245 and 106.83 ± 41.25 Hz, *p* = 0.633), respectively. However, in the control group, vorticity differed significantly between distally to the SG and the TL of the coeliac trunk (111.75 ± 36.12 Hz vs. 187.85 ± 123.78 Hz, *p* = 0.038).

## 4 Discussion

This study provides insights into hemodynamic differences in patients who develop a dSINE after FET procedure, compared to those who do not. Specifically, significant elevations in WSS and vorticity distal to the SG in the dSINE group were observed, whereas these patterns were absent in the control group. Although mean WSS values across all locations did not differ significantly between groups, the dSINE group exhibited a more concentrated distribution of higher WSS distally. These findings suggest that WSS may serve as early hemodynamic markers of dSINE risk.

Baseline characteristics revealed a high prevalence of chronic AD in the dSINE group, suggesting that the chronic nature of the dissection may predispose these patients to complications like dSINE. As an aortic dissection transitions from an acute to a chronic phase, the aortic wall undergoes significant structural changes, including increased fibrosis and calcification, leading to wall stiffening and reduced elasticity and resilience (21). In this study, the aortic area at the distal landing zone was significantly larger in the dSINE group, mainly within the FL, also indicating structural vulnerabilities that could predispose the aorta to further dilation and complications. Due to the complexity of accurately assessing oversizing in dissections the SG-R_EC_, a parameter for the remaining SG expansion potential, was applied to evaluate the relationship between SG size and TL. The greater decrease in SG-R_EC_ over time in the dSINE group indicates a greater SG expansion, which may be associated with elevated mechanical stress at the distal landing zone and play a role in dSINE development. In this cohort, dSINE occurred after an average of 2.84 ± 2.3 years post-FET, consistent with previous studies underscoring the delayed onset of this complication and the importance of long-term monitoring (6).

CFD simulations revealed distinct differences in flow pattern at the SG end and distal aorta, between the two groups. The dSINE group showed more disturbed and heterogeneous flow, indicating a higher risk of post-surgical flow instability that could contribute to long-term complications. High vorticity typically occurs in regions where the fluid motion is complex, such as around the SG edges or near areas of aortic curvature, where blood flow may experience abrupt changes in direction and velocity (12, 22). In the dSINE group, elevated vorticity values aligned with increased WSS in the distal aorta, creating a unique pattern not observed in the control group. This distal rise in vorticity prior to dSINE development may indicate underlying flow disturbances and turbulence, potentially exacerbating mechanical strain on the intimal layer and increasing the likelihood of new tear formation (23).

Although mean WSS values were similar between the dSINE and control groups, a distinct pattern was observed in the dSINE group, marked by a sharp increase in WSS from the SG area to the distal aorta. This rise in WSS, combined with higher concentrations distally, indicates localized hemodynamic stress specific to patients at risk of developing dSINE. In contrast, the control group displayed smoother transitions with distributed WSS, more uniform flow velocity, and reduced vorticity, suggesting that an intact hemodynamic coupling between parameters may protect against distal injury.

Our observations are in line with prior reports showing that high WSS at the distal landing zone of standard grafts coincides with subsequent intimal tears (24). Our findings also highlight that WSS distribution patterns and changes, rather than average WSS alone, may be key indicators of hemodynamic vulnerability. This WSS distribution may impose mechanical strain on the already weakened intimal layer, particularly since the distal landing zone of the SG is often positioned in a diseased part of the aorta, possibly leading to further intimal injury and new entry tear formation (25). Local lumen narrowing and pressure gradients have similarly been linked to increased shear and intimal injury (26), while morphological studies described the “inverted pyramid” configuration distal to the stent, associated with elevated WSS and distal TL dilation before dSINE occurs (27). Previous combined structural–flow analyses have suggested that oversizing leads to concentrated circumferential WSS at the SG edge, whereas undersizing results in disturbed flow, distal velocity acceleration, and focal WSS hotspots. Both mechanisms, however, may contribute to the development of dSINE (28). Our results extend these concepts by demonstrating that in patients with dSINE, elevated velocity, vorticity, and WSS were consistently co-localized distally to the SG end. This indicates that flow acceleration translates into rotational patterns and shear amplification, creating a hostile biomechanical environment at precisely the transition between prosthetic and native aorta. Importantly, this hostile hemodynamic profile coincided with a greater decrease in SG-REC over time, indicating that progressive stent graft expansion may have reinforced stress transfer to the distal aorta. The spatial overlap between this adverse hemodynamic condition and the region where dSINE was subsequently identified supports the hypothesis that elevated mechanical strain predisposes the distal landing zone to intimal failure.

In addition, surgical and anatomical factors, such as larger distal aortic diameters, persistent false lumen, SG design, length and position and angulation at the landing zone, may further contribute to disturbed flow patterns and locally increased WSS, emphasizing the complex interplay between anatomical configuration and hemodynamic stress.

Our findings emphasize the need to incorporate both clinical and hemodynamic parameters, such as those derived from CFD simulations, into the risk stratification of patients after FET. Monitoring WSS in the distal aorta post-FET may enable earlier identification of high-risk patients, guiding more tailored surveillance or intervention strategies. Given the resource-intensive nature of CFD, its use may be best directed toward patients with anatomical characteristics previously associated with increased dSINE risk. Furthermore, based on our results, we recommend a closer follow-up in patients exhibiting elevated WSS distal to the SG to detect early signs of dSINE and prevent adverse outcomes.

Given that this pattern was detected prior to dSINE development, WSS distribution could serve as a predictive marker. The stable WSS profile, with a gradual transition between the SG and distal regions, seen in the control group further supports the uniqueness of this distal WSS concentration as a potential risk factor. While this study cannot establish causality, the overlap between CFD-predicted hotspots and the actual site of complication strengthens the potential of CFD to identify high-risk regions prior to clinical manifestation.

### 4.1 Study limitations

This study is limited by assumptions in the fluid model and boundary conditions, including laminar blood flow, constant inflow velocity, rigid walls, steady-state simulations, a uniform inflow waveform, and zero pressure outlets, which may not fully capture transient flow conditions. While this reduced computational cost, prior work has shown that non-patient-specific inflow and simplified outlets can significantly affect WSS and pressure distributions (29, 30). Thus, our results should be interpreted as hypothesis-generating, and future studies should incorporate transient simulations with patient-specific inflow and Windkessel outlet models. Due to CTA data availability, in 2 dSINE and 3 control cases, the outlet was located at the level of the celiac trunk, approximately 10 cm downstream of the distal landing zone; although a 0 Pa condition at this plane may influence absolute values, the impact on local hemodynamics at the dSINE site is expected to be limited, but this methodological constraint should be acknowledged when interpreting the results. Direct validation with Doppler or 4D flow MRI was not feasible due to the retrospective design, but simulated velocities and wall shear stress values were within ranges reported in previous CFD and imaging studies. The applied CFD approach has already been established by our group in the context of aortic dissection and stent-graft remodeling, supporting the reliability of the present models in capturing hemodynamic mechanisms of dSINE. The higher prevalence of chronic AD in the dSINE group may have influenced results, as differences in aortic wall stiffness and remodeling between chronic and acute AD could affect hemodynamic conditions and WSS distribution. However, the rigid-wall assumption and the restriction to the patent lumen minimize the confounding effect of differences in dissection chronicity. Although the sample size is relatively small, dSINE is a rare complication, and this cohort represents one of the larger samples studied with CFD. To reduce potential selection bias, regions of interest were defined before visualizing WSS. Finally, while elevated WSS and vorticity were associated with dSINE, the lack of a formal matching process may introduce limitations, as differences in baseline characteristics such as distal aortic area and mean time between surgery and CTA used for the simulations could act as confounders. Therefore, further studies with larger, well-matched cohorts and transient, patient-specific simulations in the early postoperative phase are needed to clarify the causal role of hemodynamics in dSINE development.

### 4.2 Conclusion

This study highlights distinct hemodynamic patterns associated with the development of dSINE following FET surgery, specifically the increase in WSS distal to the SG. These localized elevations in WSS and complex flow dynamics appear to predispose certain patients to complications, making them valuable markers for identifying patients at elevated risk. The application of CFD modeling in this context enables individualized risk prediction, allowing for tailored surveillance and intervention strategies. While further research with larger patient cohorts is necessary to validate its predictive value, particularly in combination with clinical parameters, our findings emphasize the need for closer follow-up in patients with elevated WSS distal to the SG. Early detection of dSINE through tailored surveillance could enable timely interventions and improve long-term patient outcomes.

## Data Availability

The raw data supporting the conclusions of this article will be made available by the authors, without undue reservation.
